# Ketamine versus etomidate as an induction agent for tracheal intubation in critically ill adults: a Bayesian meta-analysis

**DOI:** 10.1186/s13054-024-04831-4

**Published:** 2024-02-17

**Authors:** Takatoshi Koroki, Yuki Kotani, Takahiko Yaguchi, Taisuke Shibata, Motoki Fujii, Stefano Fresilli, Mayuko Tonai, Toshiyuki Karumai, Todd C. Lee, Giovanni Landoni, Yoshiro Hayashi

**Affiliations:** 1 Department of Intensive Care Medicine, Kameda Medical Center, 929 Higashi-cho, Kamogawa, 296-8602 Japan; 2 Department of Anesthesia and Intensive Care, IRCCS San Raffaele Scientific Institute, Milan, Italy; 3 School of Medicine, Vita-Salute San Raffaele University, Milan, Italy; 4 Division of Infectious Diseases, Department of Medicine, McGill University, Montreal, QC Canada

**Keywords:** Systematic review, Meta-analysis, Ketamine, Intubation, Mortality, Intensive care units, Bayes theorem

## Abstract

**Background:**

Tracheal intubation is a high-risk intervention commonly performed in critically ill patients. Due to its favorable cardiovascular profile, ketamine is considered less likely to compromise clinical outcomes. This meta-analysis aimed to assess whether ketamine, compared with other agents, reduces mortality in critically ill patients undergoing intubation.

**Methods:**

We searched MEDLINE, Embase, and the Cochrane Library from inception until April 27, 2023, for randomized controlled trials and matched observational studies comparing ketamine with any control in critically ill patients as an induction agent. The primary outcome was mortality at the longest follow-up available, and the secondary outcomes included Sequential Organ Failure Assessment score, ventilator-free days at day 28, vasopressor-free days at day 28, post-induction mean arterial pressure, and successful intubation on the first attempt. For the primary outcome, we used a Bayesian random-effects meta-analysis on the risk ratio (RR) scale with a weakly informative neutral prior corresponding to a mean estimate of no difference with 95% probability; the estimated effect size will fall between a relative risk of 0.25 and 4. The RR and 95% credible interval (CrI) were used to estimate the probability of mortality reduction (RR < 1). The secondary outcomes were assessed with a frequentist random-effects model. We registered this study in Open Science Framework (https://osf.io/2vf79/).

**Results:**

We included seven randomized trials and one propensity-matched study totaling 2978 patients. Etomidate was the comparator in all the identified studies. The probability that ketamine reduced mortality was 83.2% (376/1475 [25%] vs. 411/1503 [27%]; RR, 0.93; 95% CrI, 0.79–1.08), which was confirmed by a subgroup analysis excluding studies with a high risk of bias. No significant difference was observed in any secondary outcomes.

**Conclusions:**

All of the included studies evaluated ketamine versus etomidate among critically ill adults requiring tracheal intubation. This meta-analysis showed a moderate probability that induction with ketamine is associated with a reduced risk of mortality.

**Graphical abstract:**

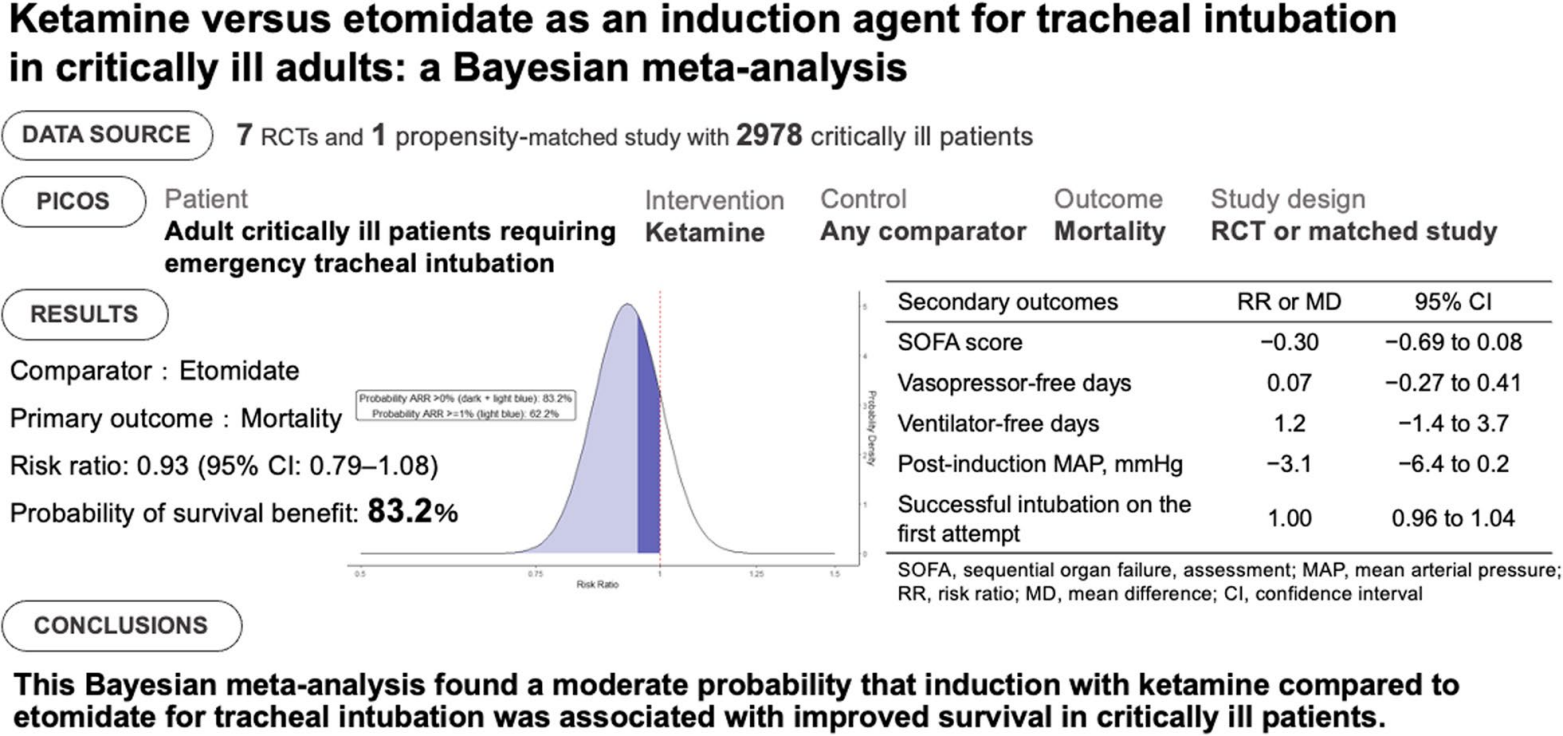

**Supplementary Information:**

The online version contains supplementary material available at 10.1186/s13054-024-04831-4.

## Background

Tracheal intubation is a high-risk procedure commonly performed in intensive care units (ICUs) [1]. Peri-intubation complications are common and are associated with an increased risk of mortality in critically ill patients [2]. Therefore, improving the quality of care in the peri-intubation period may result in better outcomes of these high-risk patients.

Rapid sequence intubation (RSI), facilitated by rapidly acting agents, is often used among critically ill patients who are deteriorating quickly. Clinical practice guidelines for RSI suggest ketamine, etomidate, and propofol as induction agents [3, 4]. Among the three drugs, propofol is an independent risk factor for cardiovascular collapse during the procedure [5], and etomidate carries a major risk of adrenal insufficiency due to inhibition of 11-beta hydroxylase in the adrenal glands [6]. Notably, a recent meta-analysis showed that etomidate was associated with significantly increased mortality in critically ill patients requiring tracheal intubation [7].

Given ketamine’s favorable hemodynamic effects and absence of the adverse effects exhibited by other agents, it may represent the optimal option in this particular context. Indeed, in a subgroup analysis of a prior meta-analysis, etomidate had numerically increased mortality when compared to ketamine although this was not statistically significant (risk ratio [RR], 1.07; 95% confidence interval [CI] 0.94–1.22; *P* = 0.30) [7].

To further the discussion on potential benefits of ketamine, we performed an updated systematic review and Bayesian meta-analysis to estimate the probability that ketamine as an induction agent would reduce mortality in critically ill patients requiring tracheal intubation.

## Methods

We performed a systematic review and meta-analysis according to the Preferred Reporting Items for Systematic Reviews and Meta-Analyses (PRISMA) guidelines [8] (see PRISMA checklist in Additional file 1) and registered the review protocol on Open Science Framework (registration link: https://osf.io/2vf79/) on March 30, 2023. Our review question was built using the PICOS (Population, Intervention, Comparison, Outcome, and Study design) framework: adult critically ill patients requiring emergency tracheal intubation (*P*); ketamine (*I*); any other comparator (*C*); all-cause mortality at the longest follow-up available (*O*); and in randomized controlled trials and matched studies (*S*).

### Search strategy and selection criteria

Two investigators independently searched MEDLINE, Embase, and Cochrane Library for relevant studies from inception to April 27, 2023. We considered eligible RCTs and matched studies comparing ketamine versus other sedatives as an induction agent for tracheal intubation in critically ill adults. We defined critically ill adults as patients requiring emergency tracheal intubation due to critical illness, regardless of where the intubation was performed (e.g., prehospital, emergency department, and intensive care unit). Critical illness was defined as a state of ill health with vital organ dysfunction and a high risk of imminent death if care is not provided [9]. We only included the studies assessing the efficacy of administrating ketamine as induction agent for critically ill adults during tracheal intubation. We excluded non-randomized trials, observational studies without matching, systematic reviews, commentaries/editorials and literature reviews, and studies not addressing our review question. The complete search strategy is provided in Additional file 2.

Two investigators independently screened eligibility based on study titles and abstracts after removing duplicates. Finally, we selected eligible studies based on full-text manuscripts. Disagreements were resolved through discussion under the supervision of one senior investigator.

### Data collection and risk of bias assessment

Two investigators independently extracted data from included studies using a standardized data collection form. We resolved disagreements by consensus or by involving a third senior author. Data such as first author, year of publication, country, study design, setting (hospital or other settings at enrollment), and primary and secondary outcomes were collected. If data ware missing for this meta-analysis or if the authors reported only short-term mortality, we contacted the first or corresponding author to request further information.

We assessed the risk of bias for randomized studies by using the Cochrane risk of bias tool for randomized trials version 2 (RoB 2) [10] and for propensity-matched studies by using risk of bias in non-randomized studies of interventions (ROBINS-I) tool [11]. We assessed the overall certainty of the evidence based on the Grading of Recommendations Assessment, Development, and Evaluation (GRADE) methodology [12]. We prepared the GRADE evidence profile tables using the GRADEpro software [13]. The presence of publication bias and small study effects on the primary outcome was investigated by visual estimation of funnel plot.

### Outcomes

The primary outcome was all-cause mortality at the longest follow-up available. The secondary outcomes included Sequential Organ Failure Assessment (SOFA) score [14], ventilator-free days at day 28, vasopressor-free days at day 28, post-induction mean arterial pressure (MAP), and successful intubation on the first attempt.

### Data analysis

For the primary outcome, we used a Bayesian random-effects meta-analysis on the RR scale. We chose a weakly informative neutral prior for mu (*N* ~ [0,0.71^2^]) corresponding to a mean estimate of no difference with a 95% probability; the estimated effect size fell between a RR of 0.25 and 4 [15]. This type of prior recognizes that: (1) There is no strong prior knowledge suggesting that ketamine is superior to other therapies; (2) there is not a demonstrable difference between most interventions in medicine; and (3) that the effect size of almost all interventions in medicine will be modest at best and so not all RRs are equally likely. For the between-study standard deviation (SD) parameter (tau), we used an informative prior based on the predictive distribution derived from hundreds of Cochrane meta-analyses that reported all-cause mortality [16]. Analysis was conducted using the bayesmeta package in R version 4.2.2 (R Foundation for Statistical Computing, Vienna, Austria) [17]. Upon model fitting, we estimated the posterior probabilities of any benefit (RR < 1) and of meaningful clinical effect (a priori defined as a 1% absolute risk reduction) based on the weighted control event rate by generalized linear mixed model and the *metaprop* package. We considered a 1% absolute risk reduction as clinically meaningful difference because, among millions of critically ill patients undergoing tracheal intubation annually in the world [18], even such a subtle difference could potentially impact thousands of lives from a public health perspective.

We performed two subgroup analyses for the primary outcome: exclusion of high-risk of bias studies and inclusion of randomized trials. A sensitivity analysis for the primary outcome was also performed using a Mantel–Haenszel random-effects model with a frequentist approach.

For the secondary outcomes, frequentist analyses were conducted using Review Manager version 5.4 [19]. We calculated RR and 95% CIs using a Mantel*–*Haenszel random-effects model. A *P* value less than 0.05 was considered statistically significant.

We also performed a trial sequential analysis (TSA) [20] for the primary outcome with a diversity-adjusted information size calculated using a two-sided alpha of 0.05, a power of 80%, an anticipated relative risk decrease of 10%, and the actual control event rate. We used the TSA Viewer software (Version 0.9 0.5 0.10 Beta. Copenhagen Trial Unit, Centre for Clinical Intervention Research, Rigshospitalet, Copenhagen, Denmark).

## Results

**Fig. 1:**
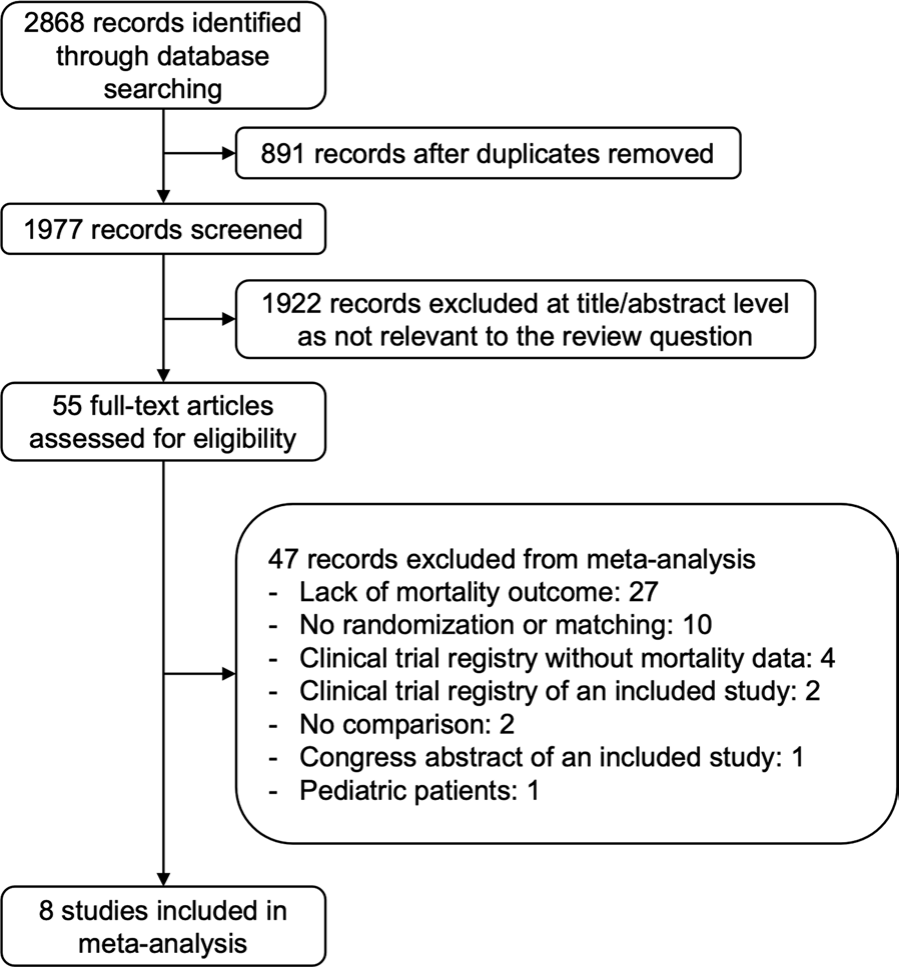
Flowchart of study selection

We included seven randomized controlled trials (RCTs) [21–27] and one propensity-matched study [28] comprising a total of 2978 critically ill adult patients (Fig. 1), with major exclusions and reasons for exclusion detailed in Additional file 13: Table S1. Despite contacting the corresponding authors of the studies without mortality data, we received no responses. The included studies were published between 2009 and 2023; four RCTs [23–26] and one propensity-matched study [28] were performed in the US, one RCT in France [21], one RCT in the Netherlands [22], and one in Thailand [27]. All but one study was single centered [22–28].

The dose of ketamine was 1–2 mg/kg [21, 23–28], except for one trial which used a combination of 0.5 mg/kg of ketamine with 0.5 mg/kg of propofol [22]. All studies used etomidate as the comparator [21–28]. The characteristics of the included studies are summarized in Table 1. Among the included studies, seven were judged at low risk of bias and one at some concerns of bias (Additional file 14: Table S2).

**Table 1:** Characteristics of included studies

First author, year	Country	No. of centers	Study design	Patients	Ketamine dose, mg/kg	Comparator	Timepoint of mortality assessment
Jabre [21]	France	65	RCT	Adult patients requiring emergency intubation	2	Etomidate	28 days
Punt [22]	Netherlands	1	RCT	Critically ill adult patients intubated in the ICU	0.5	Etomidate	28 days
Van Berkel [28]	US	1	PS-matched	Septic patients requiring prehospital intubation	1.8	Etomidate	Hospital discharge
Smischney [23]	US	1	RCT	Critically ill adults who admitted to ICU and required emergency intubation	0.5^a^	Etomidate	Hospital discharge
Driver [24]	US	1	RCT	Adult trauma patients undergoing RSI in the ED	2	Etomidate	30 days
Powers [25]	US	1	RCT	Adult patients requiring RSI	2	Etomidate	Hospital discharge
Matchett [26]	US	1	RCT	Adults requiring emergency intubation	1–2	Etomidate	28 days
Srivilaithon [27]	Thailand	1	RCT	Adult patients with suspected sepsis requiring intubation in the ED	1–2	Etomidate	28 days

*ED* Emergency department; *ICU* Intensive care unit; *PS* Propensity score; *RCT* Randomized controlled trial; and *RSI* Rapid sequence intubation

^a^Ketamine/propofol mixture = 0.5 mg/kg of ketamine plus 0.5 mg/kg of propofol

**Fig. 2:**
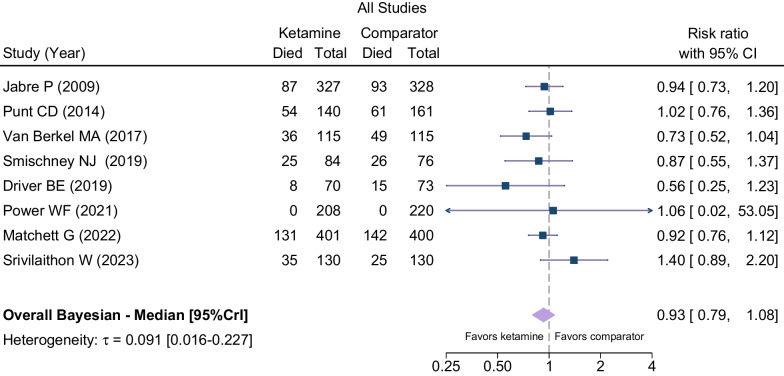
Forest plot for mortality at the longest follow-up available

**Fig. 3:**
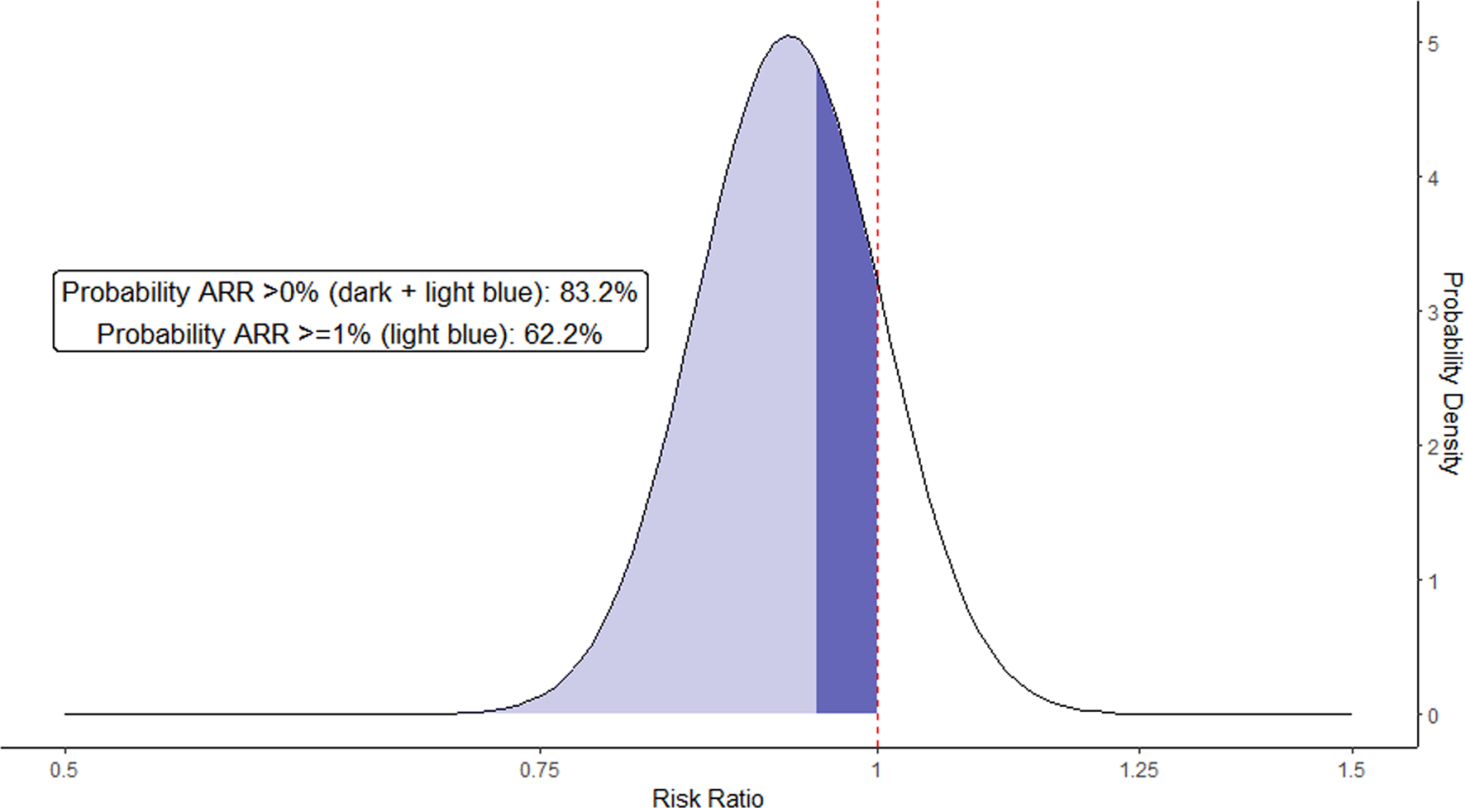
Probability density functions for combined posterior distributions of the difference in mortality in the overall population

Table 2 summarizes the outcome data. We estimated the probability that ketamine reduced mortality compared with etomidate at 83.2% (Fig. 2; 376/1475 [25%] vs. 411/1503 [27%]; RR, 0.93; 95% credible interval, 0.79–1.08) and that the probability of 1% absolute risk reduction was 62.2% (Figs. 2 and 3). The visual inspection of the funnel plot did not suggest considerable publication bias, and the TSA confirmed inconclusiveness of the findings and need for further research (Additional file 3: Fig. S1 and Additional file 4: Fig. S2). When confining the analysis to RCTs, the probability of RR < 1 was 68.6% (Table 2; Additional file 5: Fig. S3 and Additional file 6: Fig. S4). None of the included studies had a high risk of bias; therefore, a sensitivity analysis excluding studies at high risk of bias yielded the same result as the primary analysis (Table 2). A sensitivity analysis using a frequentist approach showed no significant difference between the two groups (Additional file 7: Fig. S5).

**Table 2:** Effects of ketamine on primary and secondary outcomes

Outcome	No. of studies	No. of patients	Effect measure (RR or MD)	95% CI	Probability of benefit	*I*^2^
*Primary outcome*						
Mortality at the longest follow-up available	8	2978	0.93	0.79–1.08^a^	83.2%	
Randomized controlled trials only	7	2748	0.96	0.81–1.13^a^	68.6%	
Exclusion of high risk of bias studies	8	2978	0.93	0.79–1.08^a^	83.2%	
*Secondary outcome*						
SOFA score	4	1633	− 0.30	− 0.69–0.08		0%
Vasopressor-free days (to day 28)	4	1704	0.07	− 0.27–0.41		67%
Ventilator-free days (to day 28)	4	1555	1.2	− 1.4–3.7		55%
Post-induction mean arterial pressure, mmHg	2	929	− 3.1	− 6.4–0.2		0%
Successful intubation on the first attempt	3	1204	1.00	0.96–1.04		0%

*RR* Risk ratio; *MD* Mean difference; *CI* Confidence interval; and *SOFA* Sequential organ failure assessment

^a^Indicates values are for credible interval

The SOFA score was reported in three RCTs and one matched study [21, 24, 26, 28], ventilator-free days at day 28 in four RCTs [21, 23, 24, 26], vasopressor-free days at day 28 in four RCTs [21, 22, 24, 26], post-induction MAP in two RCTs [23, 26], and successful intubation on the first attempt in three RCTs [24, 26, 27]. The pooled data detected no statistically significant between-group differences in SOFA score (MD, − 0.30; 95% CI − 0.69–0.08; *P* = 0.12; *I*^2^ = 0%), ventilator-free days at day 28 (MD, 1.2 days; 95% CI − 1.4–3.7 days; *P* = 0.38; *I*^2^ = 55%), vasopressor-free days at day 28 (MD, 0.07 days; 95% CI − 0.2–0.41 days; *P* = 0.69; *I*^2^ = 67%), post-induction MAP (MD, − 3.1 mmHg; 95% CI − 6.4–0.2 mmHg; *P* = 0.07; *I*^2^ = 0%), or successful intubation on the first attempt (RR, 1.00; 95% CI 0.96–1.04; *P* = 0.99; *I*^2^ = 0%) (see Additional file 8: Fig. S6, Additional file 9: Fig. S7, Additional file 10: Fig. S8, Additional file 11: Fig. S9, and Additional file 12: Fig. S10). The GRADE assessment is described in Additional file 15: Table S3.

## Discussion

### Key findings

This Bayesian meta-analysis of seven RCTs and one propensity-matched study found a moderate probability that ketamine as an induction agent for tracheal intubation was associated with improved survival in critically ill patients. The likelihood of survival benefits was reduced when the analysis was restricted to randomized trials alone. No statistically significant difference was observed in any secondary outcome.

### Relationship with the previous literature

Ketamine and etomidate are suggested in clinical guidelines as induction agents for RSI because of their relatively modest cardiovascular effects [3, 4]; however, few meta-analyses have focused on comparing these two agents [7, 29]. In a previous meta-analysis of randomized trials of etomidate in critically ill patients, a subgroup analysis suggested an increased mortality risk of etomidate compared with ketamine (six RCTs with 2399 patients; RR, 1.18; 95% CI 1.02–1.37) [7]. The present meta-analysis increased the sample size by adding one recent RCT [27] and one propensity-matched study [28], and found a moderate probability of mortality reduction with ketamine. However, the inconclusive TSA results and the reduced probability of benefit in the RCT subgroup leave substantial equipoise surrounding the effect of ketamine on mortality.

Hypotension is the most common peri-intubation complication in critically ill patients [2]. While etomidate was associated with less risk of post-intubation hypotension compared to ketamine in a previous meta-analysis (odds ratio, 0.53; 95% CI 0.31–0.91; *P* = 0.02) [29], the present meta-analysis showed no significant difference in post-induction MAP. The different results may be mainly attributable to different eligibility criteria. In the previous meta-analysis, no restriction on study design was placed, leading to a predominance of retrospective observational studies in the included articles [29], while the current meta-analysis selected only randomized and propensity-matched studies.

Since etomidate was the only comparator in this meta-analysis, its common adverse effect of adrenal insufficiency might have affected the mortality result. A recent meta-analysis showed that the risk of adrenal insufficiency was significantly higher in the etomidate group than the ketamine group [7]. The diagnosis of adrenal insufficiency following etomidate administration typically is made several hours to a day after induction, which is different from observation timing for SOFA score (1–3 days) [21, 24, 26, 28] and for post-induction MAP (within 1 h) [23, 26]. Therefore, adrenal insufficiency might have contributed to the mortality findings without affecting the secondary outcomes.

In addition to ketamine and etomidate, propofol is also listed among induction agents for critically ill patients [3, 4]. Although we identified no randomized or matched study that compared ketamine with propofol, a secondary analysis of a recent international large cohort study found induction with propofol as an independent risk factor for peri-intubation hemodynamic complications in critically ill patients [5].

### Implications for clinical practice and future research

One important aspect of this meta-analysis is the use of Bayesian analysis for mortality. Unlike a frequentist approach, whose conclusion always falls into a dichotomous yes or no based on the 95% CI (i.e., in this case, ketamine does not reduce mortality), Bayesian analysis can provide a more nuanced interpretation concerning the potential magnitude and direction of the treatment effect. When considering the relationship between different types of sedatives and mortality, such treatment effects may be small, which, in turn, would require large sample sizes. However, due to the urgent nature of performing tracheal intubation in critically ill patients, conducting large-scale randomized trials would prove challenging. Furthermore, the high severity and considerable heterogeneity in critically ill patients make it challenging to detect a statistically significant mortality difference attributable to a specific intervention. Given this context, we decided to perform a Bayesian analysis to allow for probabilistic interpretation about ketamine and mortality. The Bayesian approach is also beneficial from a global public health perspective. Considering the vast number of critically ill patients undergoing tracheal intubation annually [18], even a minor mortality difference could potentially have a considerable impact.

In addition, the present meta-analysis highlights the need for further investigation comparing the two key induction agents for tracheal intubation in intensive care settings. In fact, one ongoing multicenter RCT (*N* = 2324; trial registration: NCT05277896) will add important evidence to this meta-analysis.

For clinical practice, this meta-analysis cannot support a clear recommendation regarding the choice of induction agents. Recent guidelines for RSI suggested no difference regarding the effects of induction agents on mortality or hypotension [30]. However, a moderate likelihood toward decreased mortality with ketamine compared with etomidate shown in our meta-analysis may help clinical decision making when the treating clinician has experience with both drugs. Of note, etomidate is not available in several countries. For clinicians working in such countries, our study findings cannot be generalized, and future research is necessary to evaluate other induction agents with clinical equipoise.

### Strengths and limitations

This meta-analysis provides updated mortality data of ketamine compared with etomidate in critically ill patients. The inclusion of only randomized and propensity-matched studies increased the sample size and statistical power while preserving the quality of eligible studies. Most of them were judged low risk of bias, which could have improved the quality of evidence. Furthermore, Bayesian meta-analysis for mortality allowed for flexible inferences, which cannot be made with a frequentist approach. Furthermore, the addition of TSA provided another perspective to assess the robustness of the currently available evidence. The TSA suggested that not only is the impact of ketamine on mortality currently inconclusive, but also that more evidence is needed to reach a definitive conclusion.

We should acknowledge several limitations. First, among the eight studies included in this meta-analysis, etomidate was the only comparator. As a result, no conclusion is available regarding the comparison of ketamine with other induction agents. Second, peri-intubation interventions other than induction agents (e.g., opioids, neuromuscular blockades, and vasopressors) were not always standardized within each study and were heterogenous among different studies. In addition, most studies were single centered. However, randomized or matched design have minimized potential biases that may have arisen. Third, the rarity of reported psychological adverse events has hindered the evaluation of this critical outcome. Therefore, future research should investigate this relevant patient-reported outcome, particularly given that nightmare is a typical adverse event associated with ketamine use [31].

## Conclusions

This meta-analysis identified seven randomized trials and one propensity-matched study which assessed ketamine as an induction agent compared to etomidate among critically ill adults requiring tracheal intubation. We found a moderate probability that induction with ketamine, compared to etomidate, was associated with a reduced risk of mortality. Further research is required to determine the potential beneficial effects of ketamine on clinically relevant outcomes.

### Supplementary Information

**Additional file 1.** PRISMA checklist.

**Additional file 2.** Search strategy.

**Additional file 3: Fig. S1.** Funnel plots for mortality at the longest follow-up available.

**Additional file 4: Fig. S2.** Trial sequential analysis for mortality at the longest follow-up available. Alpha error = 5%, power = 80%, relative risk decrease = 10%, and diversity = 0%.

**Additional file 5: Fig. S3.** Forest plot for mortality at the longest follow-up available in randomized controlled trials.

**Additional file 6: Fig. S4.** Probability density functions for combined posterior distributions. The difference in mortality at the longest follow-up available in randomized controlled trials.

**Additional file 7: Fig. S5.** Forest plot for mortality at the longest follow-up available using a frequentist approach.

**Additional file 8: Fig. S6.** Forest plot for Sequential Organ Failure Assessment score.

**Additional file 9: Fig. S7.** Forest plot for ventilator-free days at day 28.

**Additional file 10: Fig. S8.** Forest plot for vasopressor-free days at day 28.

**Additional file 11: Fig. S9.** Forest plot for post-induction mean arterial pressure.

**Additional file 12: Fig. S10.** Forest plot for successful intubation on the first attempt.

**Additional file 13: Table S1.** Major exclusions and reasons for exclusion, in order of year of publication.

**Additional file 14: Table S2.** Risk of bias assessment of included studies.

**Additional file 15: Table S3.** GRADE evaluation.

## Data Availability

We collected the summary data from published manuscripts. The published article and its supplementary files include all the data generated or analyzed for this study. Further information is available from the corresponding authors upon reasonable request.

## Abbreviations

CI: Confidence interval
CrI: Credible interval
ICU: Intensive care unit
PRISMA: Preferred Reporting Items for Systematic Reviews and Meta-Analyses
RCT: Randomized controlled trial
RR: Risk ratio
RSI: Rapid sequence intubation
TSA: Trial sequential analysis
