# Supplementary material for: Ketamine versus etomidate as an induction agent for tracheal intubation in critically ill adults: a Bayesian meta-analysis
Source: Crit Care. 2024 Feb 17;28:48. doi: 10.1186/s13054-024-04831-4 (PMC10874027; doi:10.1186/s13054-024-04831-4)
Supplement: Supplementary file 2 — Additional file 2. Search strategy. [file 13054_2024_4831_MOESM2_ESM.docx]

# Search strategy for systematic literature review

MEDLINE

(Propensity score [MeSH] or Propensity score matching [tiab] or Propensity score analysis [tiab] or Propensity-matched [tiab] or Propensity-adjusted [tiab] or Matching score [tiab]Matched group [tiab] or Matched sample [tiab] or Matched case-control [tiab] or Case-control matching [tiab] or Matched-pair [tiab] or Pair-matching [tiab] or Pair-matched [tiab]Matched-Pair Analysis [mh] or Matched Groups [mh] or Matched Case-Control Studies [MeSH]score matc* [tiab] or score-matc* [tiab] or propensity-score [tiab] or propensity-score [tiab] or randomized controlled trial [pt] OR controlled clinical trial[pt] OR randomized controlled trials[mh] OR random allocation[mh] OR double-blind method[mh] OR single-blind method[mh] OR clinical trial[pt] OR clinical trials[mh] OR clinical trial[tw] OR latin square[tw] OR placebos[mh] OR placebo*[tw] OR random*[tw] OR research design[mh:noexp] OR follow-up studies[mh] OR prospective studies[mh] OR cross-over studies[mh] OR control*[tw] OR prospectiv*[tw] OR volunteer*[tw] OR randomized [tiab] or randomization [TIAB] or randomly [TIAB] or randomised prospective study [tiab] or post hoc analysis [tiab] or post hoc analyses [tiab] or random number table[tiab] or assigned receive [tiab] or cluster-randomized [tiab]) AND (intensive care unit [tiab] or critical care [tiab] or emergency induction [tiab] or emergency intubation [tiab] or emergency [tiab] or trauma [tiab] or Critically ill [tiab] or Critically ill patien* [tiab] or Critical Care [mh] or Critical Illness [mh] or Emergency Medical Services [mh] or Emergency Treatment [mh] or Intensive Care Units [mh]) AND (ketamine [tiab] or ketamine [mh] or Ketamine-induced [tiab] or S-Ketamine [tiab] or R-Ketamine [tiab] or CI-581 [tiab] or CI-581 [mh] or Ketanest [tiab] or ketanest [mh] or Ketaset [tiab] or ketaset [mh] or Vetalar [tiab] or Ketalar [tiab]) NOT (animal[mh] NOT human[mh] OR comment[pt] OR editorial[pt] OR meta-analysis[pt] OR practice-guideline[pt] OR review[pt])

Cochrane Library

#1 propensity score:ti,ab,kw or "propensity score matching":ti,ab,kw or "propensity score analysis":ti,ab,kw or "propensity-matched":ti,ab,kw or "propensity-adjusted":ti,ab,kw or "matching score":ti,ab,kw or "matched group":ti,ab,kw or "matched sample":ti,ab,kw or "matched case-control":ti,ab,kw or "case-control matching":ti,ab,kw or "matched-pair":ti,ab,kw or "pair-matching":ti,ab,kw or "pair-matched":ti,ab,kw or mh "matched-pair analysis" or mh "matched groups" or "matched case-control studies":ti,ab,kw or "score matc*":ti,ab,kw or "score-matc*":ti,ab,kw or "propensity-score":ti,ab,kw or "propensity-score":ti,ab,kw or "randomized controlled trial":ti,ab,kw or "controlled clinical trial":ti,ab,kw or mh "randomized controlled trials" or mh "random allocation" or mh "double-blind method" or mh "single-blind method" or "clinical trial":ti,ab,kw or mh "clinical trials" or "clinical trial":ti,ab,kw or "latin square":ti,ab,kw or mh "placebos" or "placebo*":ti,ab,kw or "random*":ti,ab,kw or mh "research design" or mh "follow-up studies" or mh "prospective studies" or mh "cross-over studies" or "control*":ti,ab,kw or "prospectiv*":ti,ab,kw or "volunteer*":ti,ab,kw or "randomized":ti,ab,kw or "randomization":ti,ab,kw or "randomly":ti,ab,kw or "randomised prospective study":ti,ab,kw or "post hoc analysis":ti,ab,kw or "post hoc analyses":ti,ab,kw or "random number table":ti,ab,kw or "assigned receive":ti,ab,kw or "cluster-randomized":ti,ab,kw

#2 "intensive care unit":ti,ab,kw or "critical care":ti,ab,kw or "emergency induction":ti,ab,kw or "emergency intubation":ti,ab,kw or "emergency":ti,ab,kw or "trauma":ti,ab,kw or "critically ill":ti,ab,kw or "critically ill patien*":ti,ab,kw or mh "Critical Care" or mh "Critical Illness" or mh "Emergency Medical Services" or mh "Emergency Treatment" or mh "Intensive Care Units"

#3 "ketamine":ti,ab,kw or "ketamine-induced":ti,ab,kw or "s-ketamine":ti,ab,kw or "r-ketamine":ti,ab,kw or mh "ci-581" or "ci-581":ti,ab,kw or mh "ketanest" or "ketanest":ti,ab,kw or mh "ketaset" or "ketaset":ti,ab,kw or "vetalar":ti,ab,kw or "ketalar":ti,ab,kw or mh "Ketamine"

#4 mh "Animals" not mh "Humans" or comment:ti,ab,kw or editorial:ti,ab,kw or meta-analysis:ti,ab,kw or practice-guideline:ti,ab,kw or review:ti,ab,kw

#5 #1 and #2 and #3 not #4

Embase

(propensity score:de OR "propensity score matching":ti,ab,kw OR "propensity score analysis":ti,ab,kw OR "propensity-matched":ti,ab,kw OR "propensity-adjusted":ti,ab,kw OR "matching score":ti,ab,kw OR "matched group":ti,ab,kw OR "matched sample":ti,ab,kw OR "matched case-control":ti,ab,kw OR "case-control matching":ti,ab,kw OR "matched-pair":ti,ab,kw OR "pair-matching":ti,ab,kw OR "pair-matched":ti,ab,kw OR "matched-pair analysis":de OR "matched groups":de OR "matched case-control studies":de OR "score matc*":ti,ab,kw OR "score-matc*":ti,ab,kw OR "propensity-score":ti,ab,kw OR "propensity-score":ti,ab,kw OR "randomized controlled trial":it OR "controlled clinical trial":it OR "randomized controlled trials":de OR "random allocation":de OR "double-blind method":de OR "single-blind method":de OR "clinical trial":it OR "clinical trials":de OR "clinical trial":ti,ab,kw OR "latin square":ti,ab,kw OR "placebos":de OR "placebo*":ti,ab,kw OR "random*":ti,ab,kw OR "research design":de NOT "exp" OR "follow-up studies":de OR "prospective studies":de OR "cross-over studies":de OR "control*":ti,ab,kw OR "prospectiv*":ti,ab,kw OR "volunteer*":ti,ab,kw OR "randomized":ti,ab,kw OR "randomization":ti,ab,kw OR "randomly":ti,ab,kw OR "randomised prospective study":ti,ab,kw OR "post hoc analysis":ti,ab,kw OR "post hoc analyses":ti,ab,kw OR "random number table":ti,ab,kw OR "assigned receive":ti,ab,kw OR "cluster-randomized":ti,ab,kw) AND ("intensive care unit":ti,ab,kw OR "critical care":ti,ab,kw OR "emergency induction":ti,ab,kw OR "emergency intubation":ti,ab,kw OR "emergency":ti,ab,kw OR "trauma":ti,ab,kw OR "critically ill":ti,ab,kw OR "critically ill patien*":ti,ab,kw OR "critical care":de OR "critical illness":de OR "emergency medical services":de OR "emergency treatment":de OR "intensive care units":de) AND ("ketamine":ti,ab,kw OR "ketamine-induced":ti,ab,kw OR "s-ketamine":ti,ab,kw OR "r-ketamine":ti,ab,kw OR "ci-581":ti,ab,kw OR "ci-581":de OR "ketanest":ti,ab,kw OR "ketanest":de OR "ketaset":ti,ab,kw OR "ketaset":de OR "vetalar":ti,ab,kw OR "ketalar":ti,ab,kw OR "ketamine":de) NOT (animal:de NOT human:de OR comment:it OR editorial:it OR meta-analysis:it OR practice-guideline:it OR review:it)
