# Supplementary figures and images for: Ketamine versus etomidate as an induction agent for tracheal intubation in critically ill adults: a Bayesian meta-analysis
Source: Crit Care. 2024 Feb 17;28:48. doi: 10.1186/s13054-024-04831-4 (PMC10874027; doi:10.1186/s13054-024-04831-4)

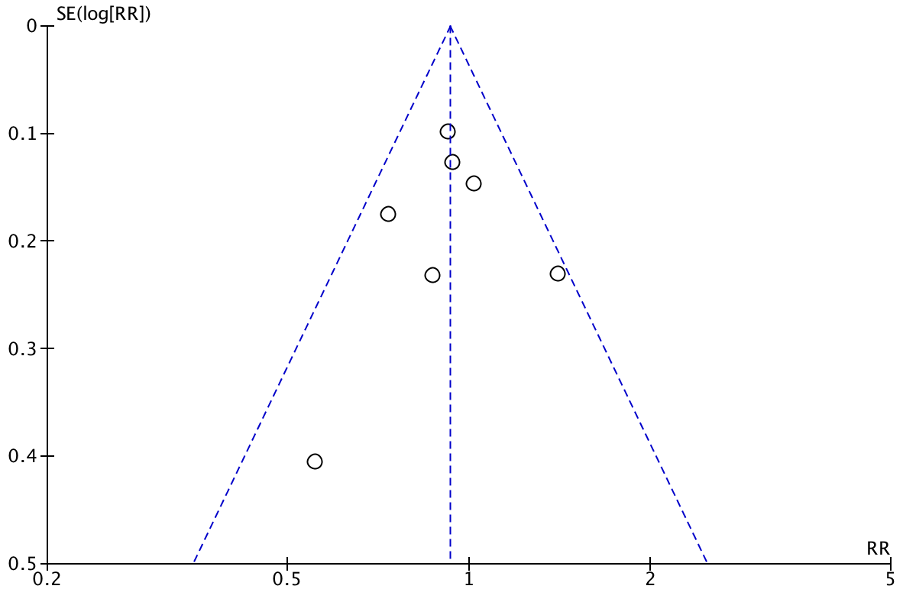

Supplement: Supplementary file 3 — Additional file 3: Fig. S1. Funnel plots for mortality at the longest follow-up available. [file 13054_2024_4831_MOESM3_ESM.png]

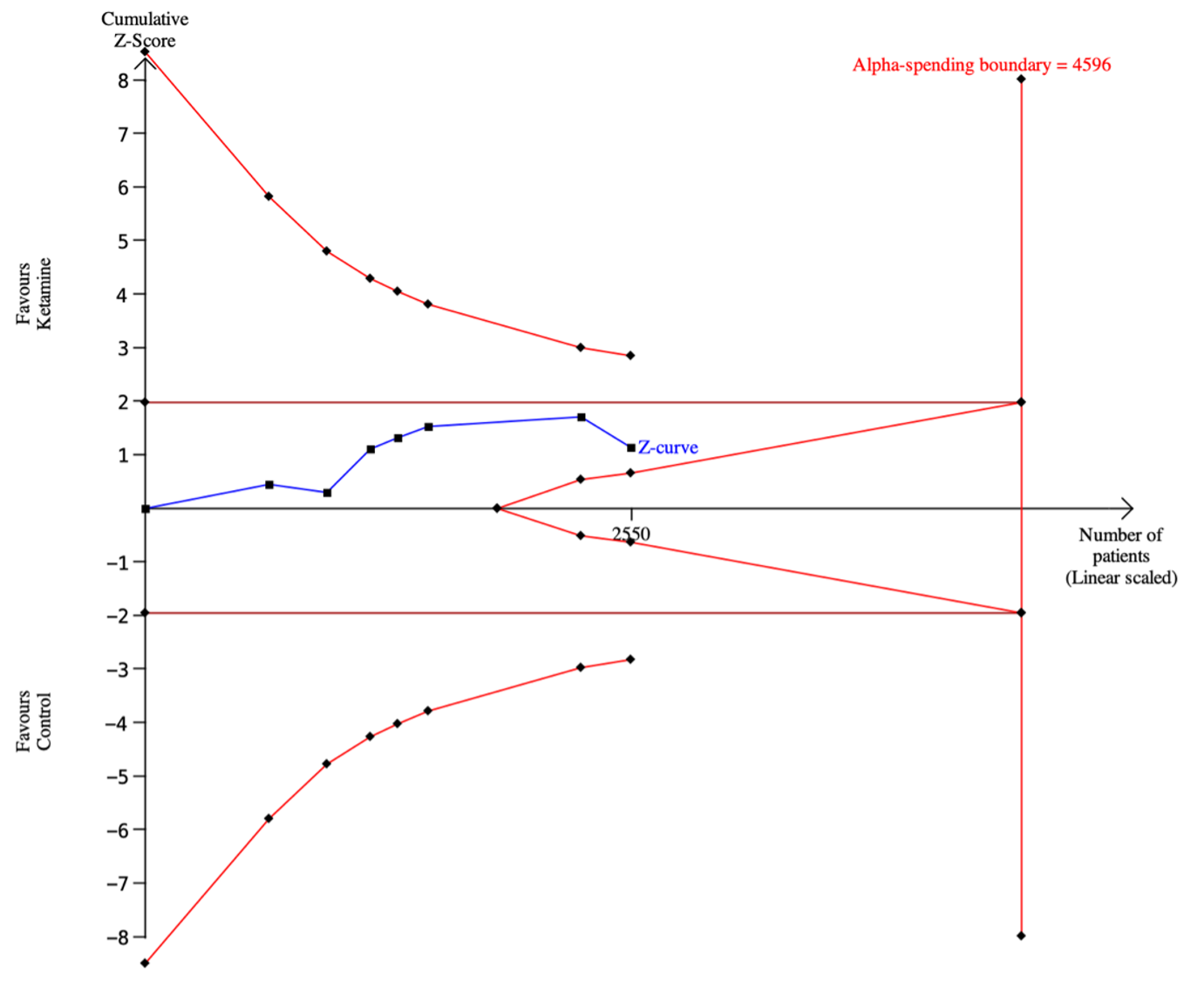

Supplement: Supplementary file 4 — Additional file 4: Fig. S2. Trial sequential analysis for mortality at the longest follow-up available. Alpha error = 5%, power = 80%, relative risk decrease = 10%, and diversity = 0%. [file 13054_2024_4831_MOESM4_ESM.png]

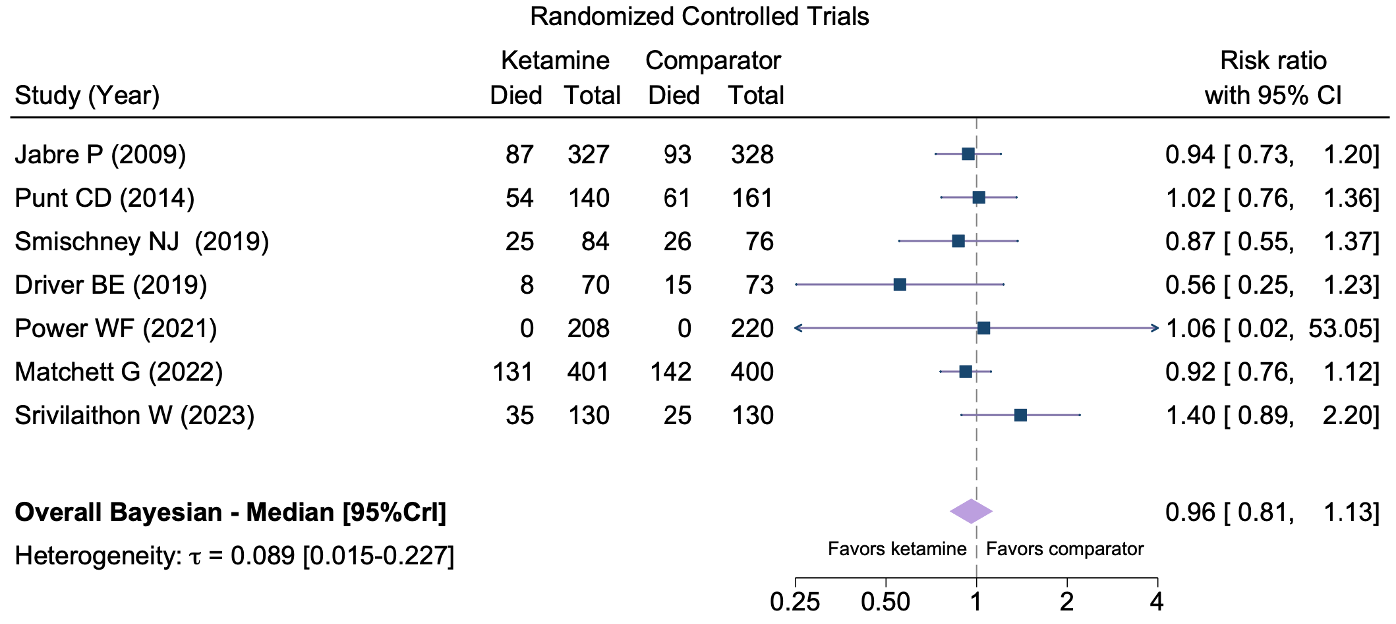

Supplement: Supplementary file 5 — Additional file 5: Fig. S3. Forest plot for mortality at the longest follow-up available in randomized controlled trials. [file 13054_2024_4831_MOESM5_ESM.png]

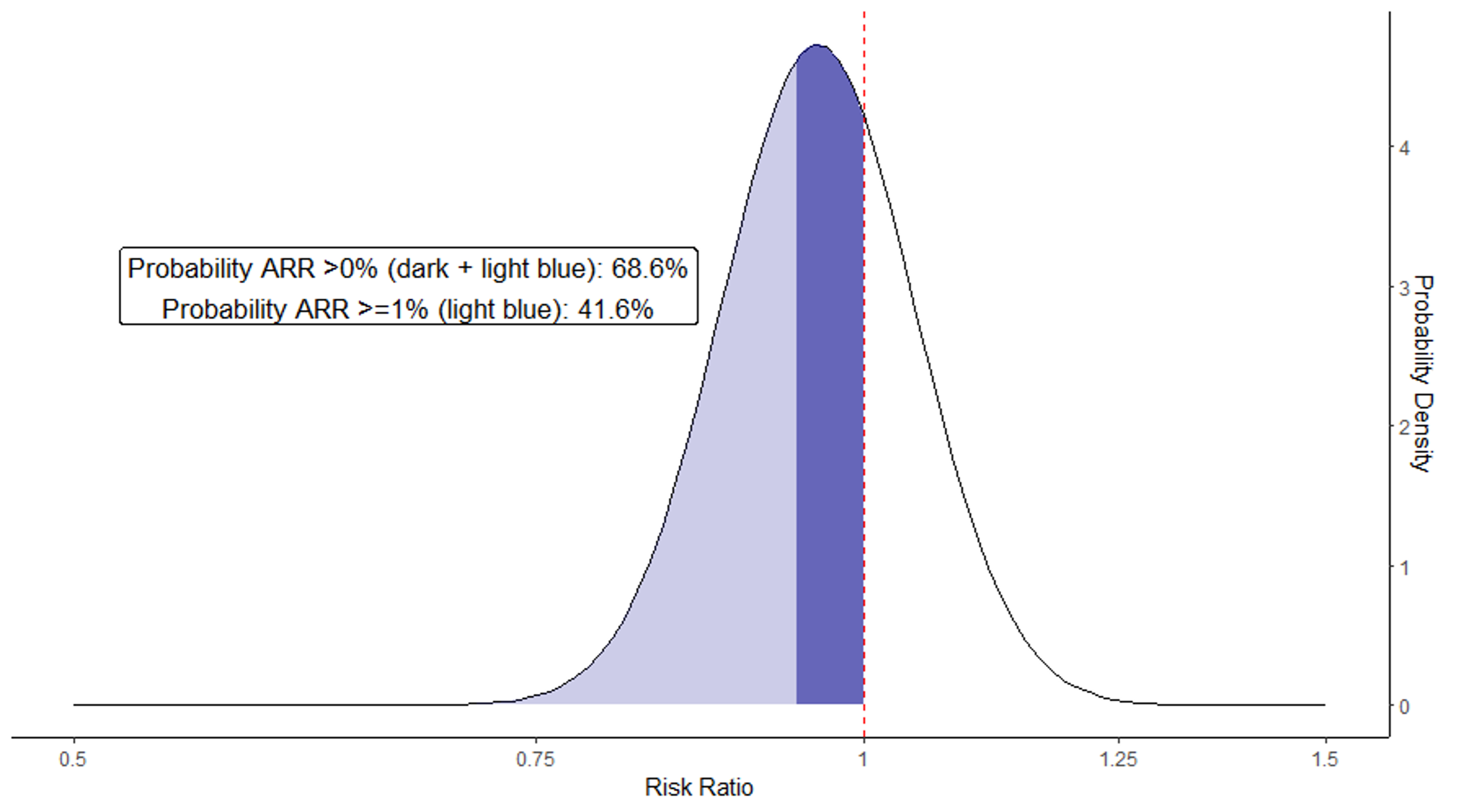

Supplement: Supplementary file 6 — Additional file 6: Fig. S4. Probability density functions for combined posterior distributions. The difference in mortality at the longest follow-up available in randomized controlled trials. [file 13054_2024_4831_MOESM6_ESM.png]

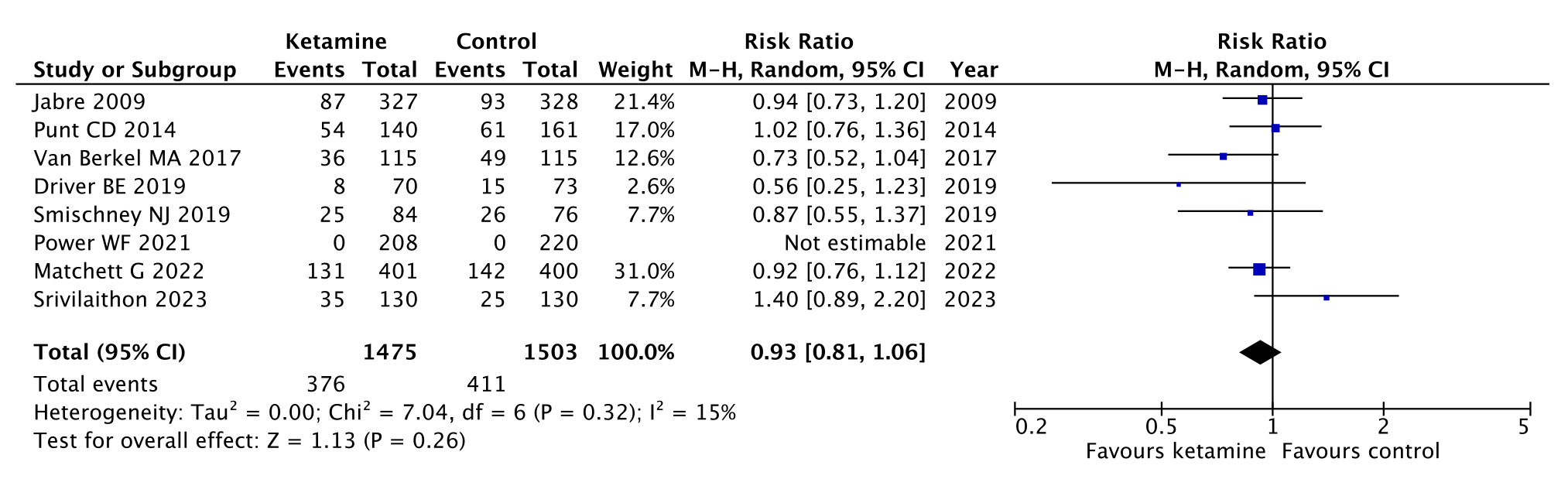

Supplement: Supplementary file 7 — Additional file 7: Fig. S5. Forest plot for mortality at the longest follow-up available using a frequentist approach. [file 13054_2024_4831_MOESM7_ESM.png]

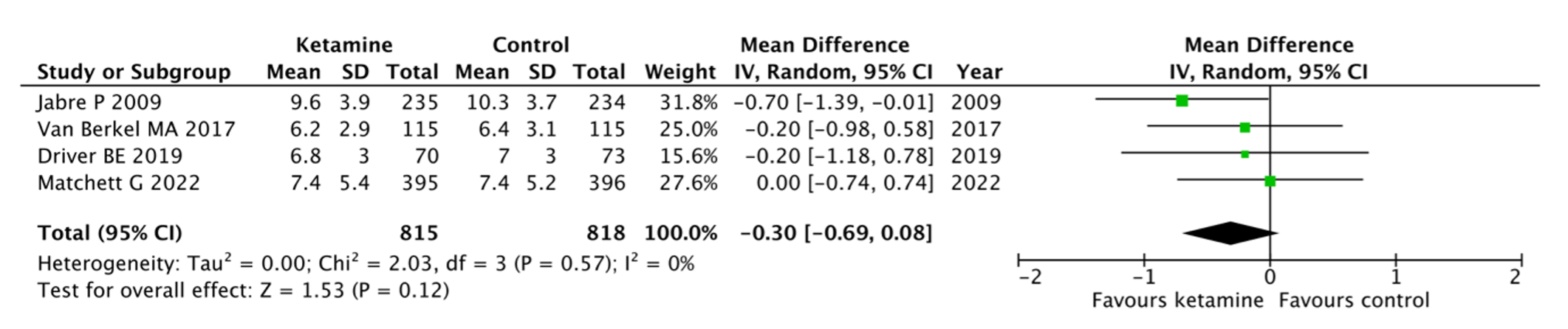

Supplement: Supplementary file 8 — Additional file 8: Fig. S6. Forest plot for Sequential Organ Failure Assessment score. [file 13054_2024_4831_MOESM8_ESM.png]

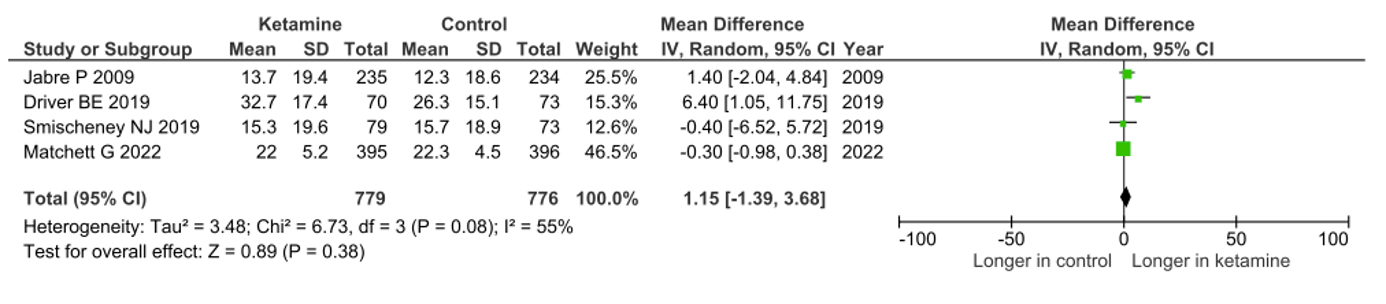

Supplement: Supplementary file 9 — Additional file 9: Fig. S7. Forest plot for ventilator-free days at day 28. [file 13054_2024_4831_MOESM9_ESM.png]

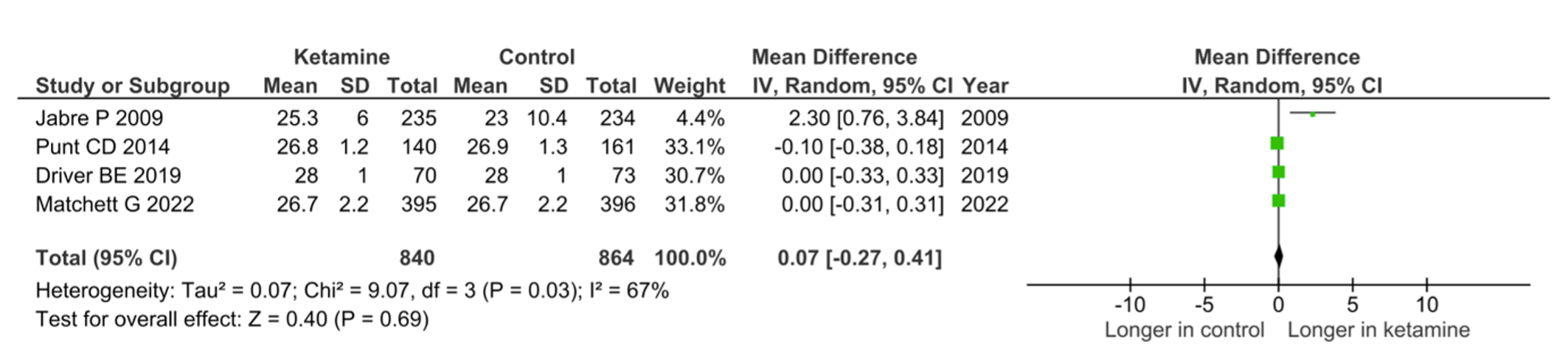

Supplement: Supplementary file 10 — Additional file 10: Fig. S8. Forest plot for vasopressor-free days at day 28. [file 13054_2024_4831_MOESM10_ESM.png]

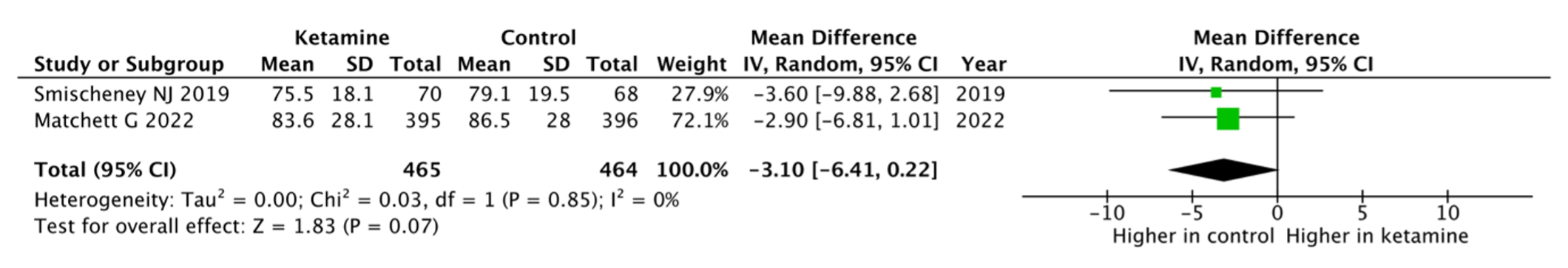

Supplement: Supplementary file 11 — Additional file 11: Fig. S9. Forest plot for post-induction mean arterial pressure. [file 13054_2024_4831_MOESM11_ESM.png]

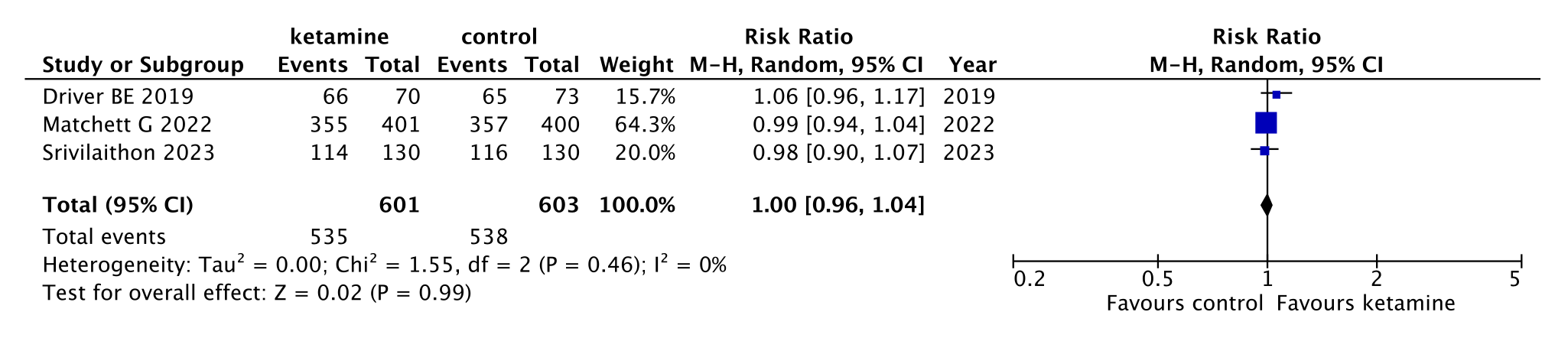

Supplement: Supplementary file 12 — Additional file 12: Fig. S10. Forest plot for successful intubation on the first attempt. [file 13054_2024_4831_MOESM12_ESM.png]
