# Supplementary material for: Ketamine versus etomidate as an induction agent for tracheal intubation in critically ill adults: a Bayesian meta-analysis
Source: Crit Care. 2024 Feb 17;28:48. doi: 10.1186/s13054-024-04831-4 (PMC10874027; doi:10.1186/s13054-024-04831-4)
Supplement: Supplementary file 13 — Additional file 13: Table S1. Major exclusions and reasons for exclusion, in order of year of publication. [file 13054_2024_4831_MOESM13_ESM.docx]

# Table S1. Major exclusions and reasons for exclusion, in order of year of publication.

| Author, year | Reason for exclusion |
| --- | --- |
| White PF, 1982 [1] | Lack of mortality data |
| Young S, 1999 [2] | Pediatric patients |
| Sivilotti MLA, 2002 [3] | Lack of mortality data |
| Jabre P, 2009 [4] | Congress abstract of an included study |
| Cinar O, 2010 [5] | Lack of mortality data |
| Sibley A, 2011 [6] | No comparison |
| Strecker N, 2011 [7] | Lack of mortality data |
| Kropt JA, 2012 [8] | Lack of mortality data |
| Kadish J, 2013 [9] | Lack of mortality data |
| McKinney C, 2013 [10] | Lack of mortality data |
| Van Berkel M, 2013 [11] | No randomization or matching |
| Lobo-Valbuena B, 2014 [12] | No randomization or matching |
| Patanwala AE, 2014 [13] | Lack of mortality data |
| Pfaff C, 2014 [14] | No randomization or matching |
| Manickam VS, 2015 [15] | Lack of mortality data |
| Okubo M, 2015 [16] | Lack of mortality data |
| Van Berkel M, 2015 [17] | Lack of mortality data |
| Baradari AG, 2016 [18] | Lack of mortality data |
| Upchurch CP, 2016 [19] | Lack of mortality data |
| Alkhouri H 2017 [20] | Lack of mortality data |
| Ishimaru T, 2018 [21] | Lack of mortality data |
| Jo SW, 2018 [22] | No randomization or matching |
| Baekgaard 2019 [23] | No randomization or matching |
| Ishimaru T, 2019 [24] | Lack of mortality data |
| Kang V, 2019 [25] | No randomization or matching |
| Nakajima S, 2019 [26] | Lack of mortality data |
| Shannon B, 2019 [27] | Lack of mortality data |
| April MD 2020 [28] | No randomization or matching |
| Farrell NM, 2020 [29] | Lack of mortality data |
| Mohr NM, 2020 [30] | Lack of mortality data |
| Godwin HT, 2020A [31] | Lack of mortality data |
| Godwin HT, 2020B [32] | Lack of mortality data |
| Bakhsh A, 2021 [33] | Lack of mortality data |
| Stanke L, 2021 [34] | No randomization or matching |
| Wan C, 2021 [35] | Lack of mortality data (after matching) |
| Ali H, 2021 [36] | Lack of mortality data |
| Freeman J, 2022 [37] | Lack of mortality data |
| Hidalgo DC, 2022 [38] | No randomization or matching |
| Kunkel S, 2022 [39] | Lack of mortality data |
| Martinez Barrios E, 2022 [40] | No comparison |
| Umana E, 2022 [41] | No randomization or matching |
| IRCT201103114365N7 [42] | Clinical trial registry without mortality data |
| NCT00440102 [43] | Clinical trial registry without mortality data |
| NCT01823328 [44] | Clinical trial registry without mortality data |
| NCT02105415 [45] | Clinical trial registry of an included study |
| NCT04120870 [46] | Clinical trial registry without mortality data |
| TCTR20210213001 [47] | Clinical trial registry of an included study |

**References**

1. White PF. Comparative evaluation of intravenous agents for rapid sequence induction--thiopental, ketamine, and midazolam. Anesthesiology. 1982;57:279–84.

2. Young S. Comparing the use of ketamine and midazolam in emergency settings. Emerg Nurse. 1999;7:27–30.

3. Sivilotti MLA, Filbin MR, Murray HE, Slasor P, Walls RM. Does the sedative agent facilitate emergency rapid sequence intubation? Acad Emerg Med. 2003;10:612–20.

4. Jabre P, Combes X, Ricard-Hibon A, Mirat P, Cibien JF, Bourzeix C, et al. Etomidate versus ketamine for rapid sequence intubation in acutely ill patients: a multicenter randomized controlled trial. Crit Care. 2009;13:1–2.

5. Abstracts of the Society of Critical Care Medicine 40th Critical Care Congress. San Diego, California, USA. January 15-19, 2011. Crit Care Med. 2010;38:A1–306.

6. Sibley A, Mackenzie M, Bawden J, Anstett D, Villa-Roel C, Rowe BH. A prospective review of the use of ketamine to facilitate endotracheal intubation in the helicopter emergency medical services (HEMS) setting. Emerg Med J. 2011;28:521–5.

7. Sepsis and multiorgan dysfunction. Weimar Sepsis Update 2011- Bridging the Gap. Abstracts of the 5th International Congress of the German Sepsis Society. September 7-11, 2011. Weimar, Germany. Infection. 2011;39 Suppl 2:S95-151.

8. Kropf JA, Grossman, Genzlinger MA, Stoltzfus J, Stehly CD. 328 Ketamine versus Etomidate for Rapid Sequence Intubation in Traumatically Injured Patients: An Exploratory Study. Ann Emerg Med. 2012;60:S117.

9. Kadish J, Salen P, Genzlinger M, Grossman M, Stoltzfus J, Kropf J. An Exploratory Study Comparing Ketamine v. Etomidate for Rapid Sequence Intubation in Traumatically Injured Patients. Ann Emerg Med. 2013;62:S30.

10. McKinney C, Patanwala A, Sakles J, Erstad B. 61: EFFECT OF ETOMIDATE VERSUS KETAMINE ON INTUBATION SUCCESS IN THE EMERGENCY DEPARTMENT. Crit Care Med. 2012;40:1.

11. Van Berkel M, Exline M, Mount K, Ryder L, Philips G, Ali N, et al. 902: Clinical hypotension with etomidate or ketamine used for intubation in a medical intensive care unit. Crit Care Med. 2013;41:A226.

12. Abstracts from the 27th annual congress, ESICM LIVES 2014, 27 September--1 October, 2014, Barcelona, Spain. Intensive Care Med. 2014;40 Suppl 1:1–308.

13. Patanwala AE, McKinney CB, Erstad BL, Sakles JC. Retrospective analysis of etomidate versus ketamine for first-pass intubation success in an academic emergency department. Acad Emerg Med. 2014;21:87–91.

14. Pfaff C, Harger N, Droege C, Ernst N, Mueller E. 1096: EVALUATION OF HEMODYNAMIC AND ADVERSE EFFECTS OF KETAMINE VERSUS ETOMIDATE FOR RSI IN TRAUMA. Crit Care Med. 2014;42:A1624.

15. Manickam VS, Mohanasundaram P. Ketofol versus fentofol as induction agents for endotracheal intubation in emergency room. Int J Emerg Med. 2015;8:1–1.

16. Okubo M, Gibo K, Hagiwara Y, Watase H, Nakayama Y, Hasegawa K. 108 Ketamine as an Induction Agent in Emergency Department Intubation: Prospective Observational Multi-Center Study in Japan. Ann Emerg Med. 2015;66:S38.

17. Van Berkel M, McCauley M, Exline M, Mount K, Ryder L, Phillips G, et al. 601: PROPENSITY MATCHED ANALYSIS COMPARING HYPOTENSION BETWEEN ETOMIDATE AND KETAMINE IN SEPTIC PATIENTS. Crit Care Med. 2015;43:152.

18. Gholipour Baradari A, Firouzian A, Zamani Kiasari A, Aarabi M, Emadi SA, Davanlou A, et al. Effect of Etomidate Versus Combination of Propofol-Ketamine and Thiopental-Ketamine on Hemodynamic Response to Laryngoscopy and Intubation: A Randomized Double Blind Clinical Trial. Anesth Pain Med. 2016;6:e30071.

19. 2016 annual meeting supplement. Acad Emerg Med. 2016;23 Suppl 1:S7–294.

20. Alkhouri H, Vassiliadis J, Murray M, Mackenzie J, Tzannes A, McCarthy S, et al. Emergency airway management in Australian and New Zealand emergency departments: A multicentre descriptive study of 3710 emergency intubations. Emerg Med Australas. 2017;29:499–508.

21. 2018 annual meeting supplement. Acad Emerg Med. 2018;25 Suppl 1:S8–298.

22. Jo SW, Hwang SY, Jo IJ, Lee TR, Shin TG. Ketamine use for endotracheal intubation in severe sepsis and septic shock. Signa Vitae [Internet]. 2018 [cited 2023 Oct 12];14. Available from: https://www.researchgate.net/publication/328766711_Ketamine_use_for_endotracheal_intubation_in_severe_sepsis_and_septic_shock

23. Drægert C, Vedel A, Nilsson J, Rasmussen L, Marker S, Krag M, et al. Abstracts. Acta Anaesthesiol Scand [Internet]. 2019 [cited 2023 Oct 10];63. Available from: https://onlinelibrary.wiley.com/doi/10.1111/aas.13442

24. Ishimaru T, Goto T, Takahashi J, Okamoto H, Hagiwara Y, Watase H, et al. Association of ketamine use with lower risks of post-intubation hypotension in hemodynamically-unstable patients in the emergency department. Sci Rep. 2019;9:1–8.

25. Kang V, Sabino G, Wu S, Arends J, Jordan K. KETAMINE VS ETOMIDATE FOR RAPID SEQUENCE INTUBATION IN THE CRITICALLY ILL POPULATION. Chest. 2019;156:A204.

26. Nakajima S, Taylor K, Zimmerman LH, Collopy K, Fales C, Powers WIV. 848: HEMODYNAMIC EFFECTS OF KETAMINE VERSUS ETOMIDATE DURING RAPID SEQUENCE INTUBATION IN AN ED. Crit Care Med. 2019;47:403.

27. Shannon B, Richards J, Seigel T, Fisher D, Sankoff J, Wilcox S. 894: DOSING OF RAPID SEQUENCE INTUBATION MEDICATIONS IN THE EMERGENCY DEPARTMENT. Crit Care Med. 2019;47:426.

28. April MD, Arana A, Schauer SG, Davis WT, Oliver JJ, Fantegrossi A, et al. Ketamine Versus Etomidate and Peri-intubation Hypotension: A National Emergency Airway Registry Study. Acad Emerg Med. 2020;27:1106–15.

29. Farrell NM, Killius K, Kue R, Langlois BK, Nelson KP, Golenia P. A Comparison of Etomidate, Ketamine, and Methohexital in Emergency Department Rapid Sequence Intubation. J Emerg Med. 2020;59:508–14.

30. Mohr NM, Pape SG, Runde D, Kaji AH, Walls RM, Brown CA. Etomidate Use Is Associated With Less Hypotension Than Ketamine for Emergency Department Sepsis Intubations: A NEAR Cohort Study. Acad Emerg Med. 2020;27:1140–9.

31. 2020 Annual Meeting Supplement. Acad Emerg Med. 2020;27 Suppl 1:S7–335.

32. Godwin HT, Fix ML, Baker O, Madsen T, Walls RM, Brown CA 3rd. Emergency Department Airway Management for Status Asthmaticus With Respiratory Failure. Respir Care. 2020;65:1904–7.

33. Bakhsh A, Alnashri M, Alawami F, Aseel R, Almaghthawi M, Alrahaili G, et al. Changes in hemodynamic parameters with the use of etomidate versus ketamine induction in the emergency department. Signa Vitae. 2021;17:85–92.

34. Stanke L, Nakajima S, Zimmerman LH, Collopy K, Fales C, Powers W 4th. Hemodynamic Effects of Ketamine Versus Etomidate for Prehospital Rapid Sequence Intubation. Air Med J. 2021;40:312–6.

35. Wan C, Hanson AC, Schulte PJ, Dong Y, Bauer PR. Propofol, Ketamine, and Etomidate as Induction Agents for Intubation and Outcomes in Critically Ill Patients: A Retrospective Cohort Study. Crit Care Explor. 2021;3:e0435.

36. Ali H, Abdelhamid BM, Hasanin AM, Amer AA, Rady A. Ketamine-based Versus Fentanyl-based Regimen for Rapid-sequence Endotracheal Intubation in Patients with Septic Shock: A Randomised Controlled Trial. Rom J Anaesth Intensive Care. 2021;28:98–104.

37. Freeman J, Alkhouri H, Knipp R, Fogg T, Gillett M. Mapping haemodynamic changes with rapid sequence induction agents in the emergency department. Emerg Med Australas. 2022;34:237–43.

38. Hidalgo DC, Amin V, Hukku A, Kutlu K, Rech MA. Etomidate Use for Rapid Sequence Intubation Is Not Associated With Nosocomial Infection. J Pharm Pract. 2022;35:383–7.

39. Kunkel S, Lenz T. Hemodynamics in Helicopter Emergency Medical Services (HEMS) Patients Undergoing Rapid Sequence Intubation With Etomidate or Ketamine. J Emerg Med. 2022;62:163–70.

40. ESICM LIVES 2022: part 1. Intensive Care Med Exp. 2022;10:39.

41. Umana E, Foley J, Grossi I, Deasy C, O’Keeffe F, Irish Trainee Emergency Research Network (ITERN) Collaborators. National Emergency Resuscitation Airway Audit (NERAA): a pilot multicentre analysis of emergency intubations in Irish emergency departments. BMC Emerg Med. 2022;22:91.

42. IRCT201103114365N7 [Internet]. [cited 2023 Oct 12]. Available from: https://trialsearch.who.int/Trial2.aspx?TrialID=IRCT201103114365N7

43. NCT. Ketamine Versus Etomidate During Rapid Sequence Intubation: Consequences on Hospital Morbidity (KETASED) [Internet]. [cited 2023 Oct 12]. Available from: https://www.clinicaltrials.gov/study/NCT00440102

44. NCT. Ketamine Versus Etomidate for Rapid Sequence Intubation [Internet]. [cited 2023 Oct 12]. Available from: https://clinicaltrials.gov/study/NCT01823328

45. Ketamine /​ Propofol Admixture “Ketofol” at Induction in the Critically Ill Against Etomidate: KEEP PACE Trial (KEEP PACE) [Internet]. NCT02105415. [cited 2023 Oct 12]. Available from: https://clinicaltrials.gov/study/NCT02105415?tab=results

46. NCT. CTG Labs - NCBI [Internet]. Comparison of Ketamine and Etomidate During Rapid Sequence Intubation in Trauma Patients. [cited 2023 Oct 12]. Available from: https://clinicaltrials.gov/study/NCT04120870

47. TCTR20210213001 [Internet]. [cited 2023 Oct 12]. Available from: https://trialsearch.who.int/Trial2.aspx?TrialID=TCTR20210213001
