# Supplementary material for: Ketamine versus etomidate as an induction agent for tracheal intubation in critically ill adults: a Bayesian meta-analysis
Source: Crit Care. 2024 Feb 17;28:48. doi: 10.1186/s13054-024-04831-4 (PMC10874027; doi:10.1186/s13054-024-04831-4)

# Table S2. Risk of bias assessment of included studies.

ROB-2 tool for randomized controlled trials


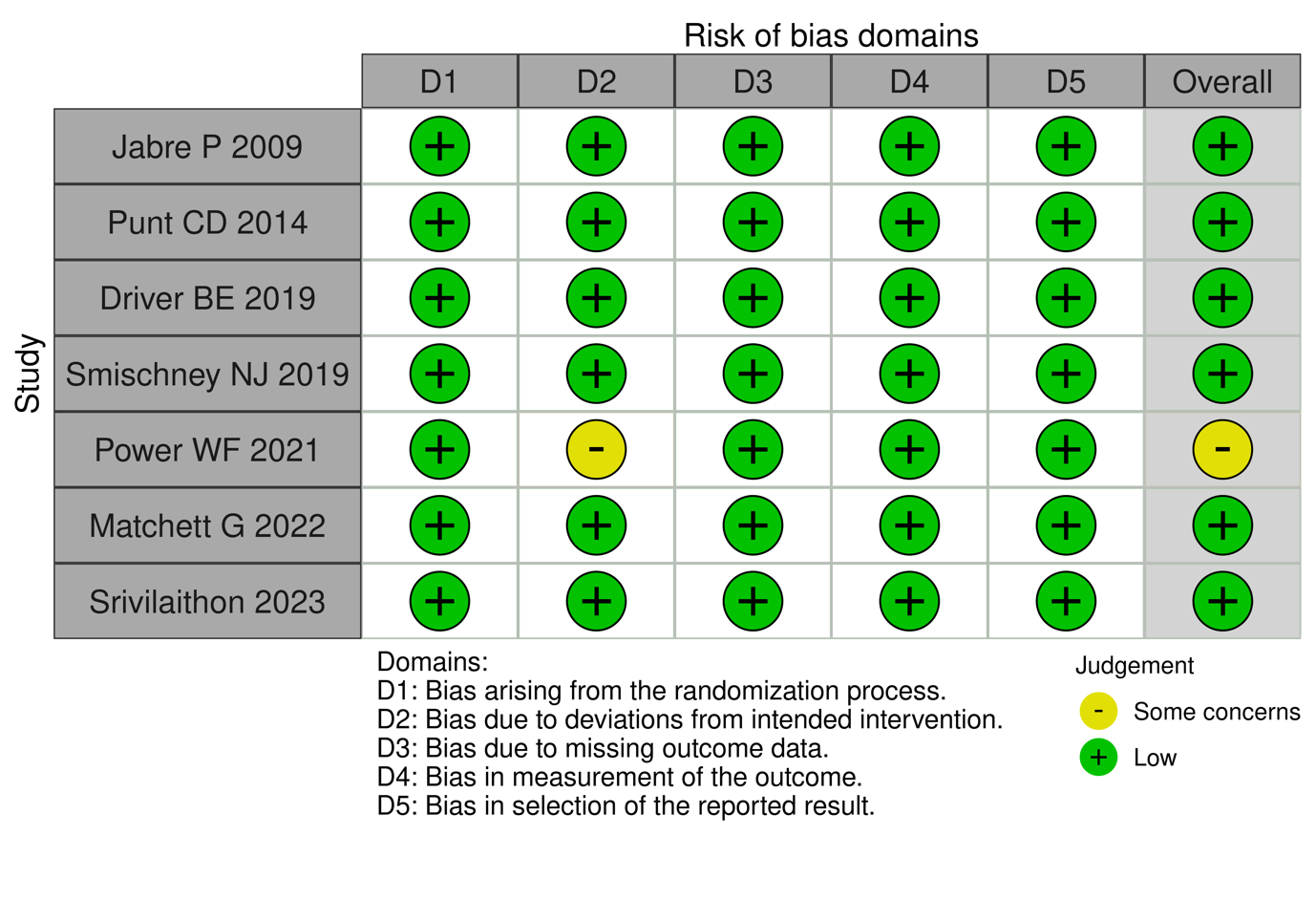


ROBINS-2 tool for non-randomized studies


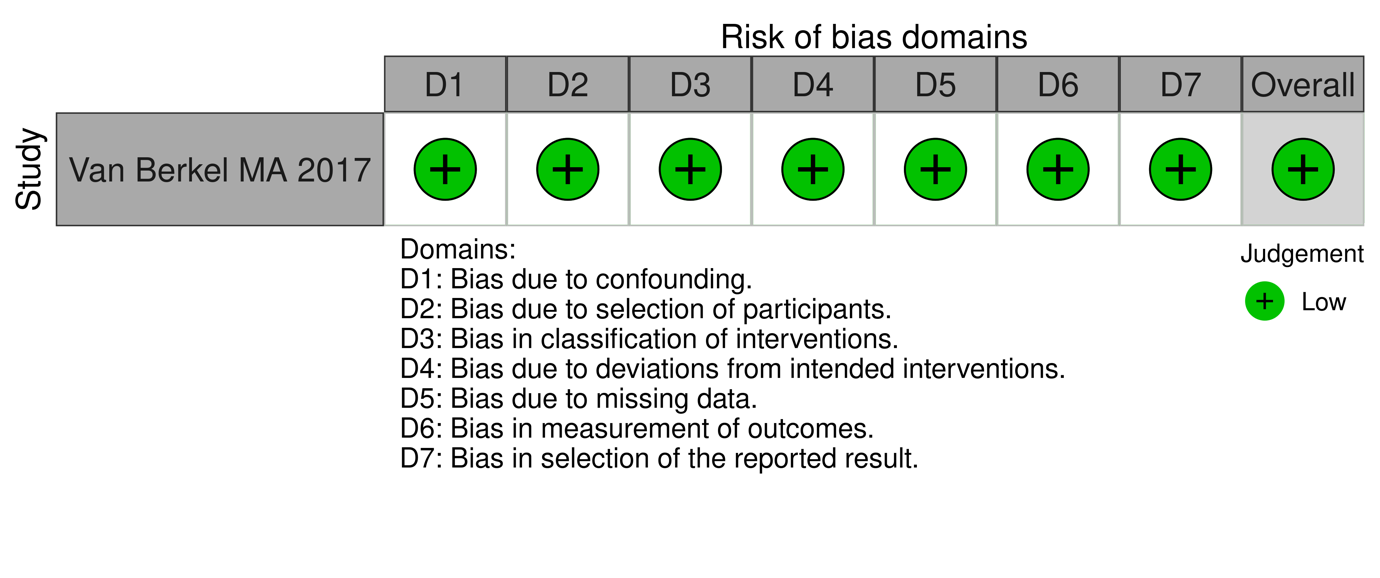

Supplement: Supplementary file 14 — Additional file 14: Table S2. Risk of bias assessment of included studies. [file 13054_2024_4831_MOESM14_ESM.docx]
