# Supplementary material for: Ketamine versus etomidate as an induction agent for tracheal intubation in critically ill adults: a Bayesian meta-analysis
Source: Crit Care. 2024 Feb 17;28:48. doi: 10.1186/s13054-024-04831-4 (PMC10874027; doi:10.1186/s13054-024-04831-4)
Supplement: Supplementary file 15 — Additional file 15: Table S3. GRADE evaluation. [file 13054_2024_4831_MOESM15_ESM.docx]

# Table S3. GRADE evaluation.

| **Certainty assessment** | | | | | | | **№ of patients** | | **Effect** | | **Certainty** | **Importance** |
| --- | --- | --- | --- | --- | --- | --- | --- | --- | --- | --- | --- | --- |
| **№ of studies** | **Study design** | **Risk of bias** | **Inconsistency** | **Indirectness** | **Imprecision** | **Other considerations** | **ketamine** | **control** | **Relative (95% CI)** | **Absolute (95% CI)** |  |  |
| **Mortality at the longest follow-up available** | | | | | | | | | | | | |
| 8 | randomised trials and an observational study | not serious | not serious | serious^a^ | not serious | none | 376/1475 (25.5%) | 411/1503 (27.3%) | **RR 0.93** (0.83 to 1.04) | **19 fewer per 1,000** (from 46 fewer to 11 more) | ⨁⨁⨁◯ Moderate | CRITICAL |
| **Sequential organ failure assessment score** | | | | | | | | | | | | |
| 4 | randomised trials and an observational study | not serious | not serious | serious^a^ | not serious | none | 815 | 818 | - | MD **0.3 lower** (0.69 lower to 0.08 higher) | ⨁⨁⨁◯ Moderate | IMPORTANT |
| **Ventilator-free days at day 28** | | | | | | | | | | | | |
| 4 | randomised trials | serious | not serious | not serious | not serious | none | 779 | 776 | - | MD **1.15 days more** (1.39 fewer to 3.68 more) | ⨁⨁⨁◯ Moderate | IMPORTANT |
| **Vasopressor-free days at day 28** | | | | | | | | | | | | |
| 4 | randomised trials | serious | not serious | not serious | not serious | none | 840 | 864 | - | MD **0.07 days more** (0.27 fewer to 0.41 more) | ⨁⨁⨁◯ Moderate | IMPORTANT |
| **Post-induction mean arterial pressure** | | | | | | | | | | | | |
| 2 | randomised trials | not serious | not serious | serious^b^ | not serious | none | 465 | 464 | - | MD **3.1 mmHg lower** (6.41 lower to 0.22 higher) | ⨁⨁⨁◯ Moderate | IMPORTANT |
| **Successful intubation on the first attempt** | | | | | | | | | | | | |
| 3 | randomized trials | serious^c^ | not serious | not serious | not serious | none | 535/601 (89.0%) | 538/603 (89.2%) | **RR 1.00** (0.96 to 1.04) | **0 fewer per 1,000** (from 36 fewer to 36 more) | ⨁⨁⨁◯ Moderate | IMPORTANT |

**CI:** confidence interval; **MD:** mean difference; **RR:** risk ratio

#### Explanations

a. Indirectness was downgraded by one level since this outcome was assessed at different timepoints among the included studies.

b. Indirectness was downgraded by one level since the assessment timing was not specified in one study.

c. The definition of this outcome was not specified in the included studies.
